# Usefulness of Matrix-Assisted Laser Desorption/Ionization-Time of Flight Mass Spectrometry in the Characterization of *Leishmania* Strains Causing Tegumentary Leishmaniasis in Bolivia versus *hsp70* Gene Sequencing

**DOI:** 10.1128/spectrum.03477-22

**Published:** 2023-01-12

**Authors:** Mary Cruz Torrico, Anna Fernández-Arévalo, Cristina Ballart, Marco Solano, Ernesto Rojas, Alba Abras, Fabiola Gonzales, Albert Arnau, Silvia Tebar, Teresa Llovet, Daniel Lozano, Eva Ariza-Vioque, Joaquim Gascón, Albert Picado, Faustino Torrico, Carmen Muñoz, Montserrat Gállego

**Affiliations:** a Facultad de Medicina, Universidad Mayor de San Simón, Cochabamba, Bolivia; b Fundación CEADES y Medio Ambiente, Cochabamba, Bolivia; c Secció de Parasitologia, Departament de Biologia, Sanitat i Medi Ambient, Facultat de Farmàcia i Ciències de l’Alimentació, Universitat de Barcelona, Barcelona, Spain; d Instituto de Salud Global de Barcelona (ISGlobal), Barcelona, Spain; e Departament de Biologia, Universitat de Girona, Girona, Spain; f Servei de Microbiologia, Hospital de la Santa Creu i Sant Pau Barcelona, Barcelona, Spain; g Departament de Genètica i Microbiologia, Universitat Autònoma de Barcelona, Bellaterra, Spain; h Institut d’Investigacions Biomèdiques August Pi i Sunyer (IDIBAPS), Barcelona, Spain; i CIBERINFEC, ISCIII-CIBER de Enfermedades Infecciosas, Instituto de Salud Carlos III; j Institut de Recerca Biomèdica Sant Pau, Barcelona, Spain; University of Edinburgh

**Keywords:** *Leishmania*, characterization, MALDI-TOF MS, *hsp70* gene sequencing, tegumentary leishmaniasis, Bolivia

## Abstract

Matrix-assisted laser desorption/ionization-time of flight mass spectrometry (MALDI-TOF MS) is a proteomic technique with proven efficiency in the identification of microorganisms, such as bacteria, fungi, and parasites. The present study aimed to evaluate the usefulness of MALDI-TOF MS for the characterization of *Leishmania* species circulating in Bolivia using *hsp70* gene sequencing as a reference technique. 55 *Leishmania* strains that were isolated from patients with tegumentary leishmaniasis were analyzed. MALDI-TOF MS identified two species of the *L. braziliensis* complex (*L. braziliensis*, *n* = 26; *L*. *braziliensis* outlier, *n* = 18), one species of the *L. guyanensis* complex (*L. guyanensis*, *n* = 1), one species of the *L. lainsoni* complex (*L. lainsoni*, *n* = 2), and two species of the L. mexicana complex (L. amazonensis, *n* = 5; and *L. garnhami*, *n* = 3). All of the strains were correctly identified at the subgenus, genus, and complex level, but 10 of them (18%) were misidentified as other species within the same complex by the *hsp70* gene sequencing, with 7 of these corresponding to possible hybrids. Thus, one *L. braziliensis* corresponded to *L. peruviana*, two *L. braziliensis* corresponded to *L. braziliensis*/*L. peruviana* possible hybrids, two L. amazonensis corresponded to L. mexicana, and three *L. garnhami* and two L. amazonensis corresponded to L. mexicana/L. amazonensis possible hybrids. Accordingly, MALDI-TOF MS could be used as an alternative to molecular techniques for the identification of *Leishmania* spp., as it is low cost, simple to apply, and able to quickly produce results. In Bolivia, its application would allow for the improvement of the management of patient follow-ups, the updating of the epidemiological data of the *Leishmania* species, and a contribution to the control of tegumentary leishmaniasis.

**IMPORTANCE** The objective of the study was to evaluate the usefulness of MALDI-TOF MS for the characterization of *Leishmania* species circulating in Bolivia, in comparison with the sequencing of the *hsp70* gene. In our study, all of the isolates could be identified, and no misidentifications were observed at the complex level. Although the equipment implies a high initial investment in our context, MALDI-TOF MS can be used in different areas of microbiology and significantly reduces the cost of testing. Once the parasite culture is obtained, the technique quickly yields information by accessing a free database that is available online. This would allow for the improvement of the management of patients and follow-ups, the updating of the epidemiological data of the species, and a contribution to the control of tegumentary leishmaniasis in Bolivia. Likewise, it can be used to determine a specific treatment to be given, according to the causal species of *Leishmania*, when there are protocols in this regard in the area.

## INTRODUCTION

Leishmaniasis is caused by protozoa of the genus *Leishmania*, which includes at least 22 species that are pathogenic to humans and are grouped within the subgenera *Leishmania* and *Viannia*. In the New World, 15 species of *Leishmania* with different patterns of tropism (visceral leishmaniasis [VL], cutaneous leishmaniasis [CL], and mucosal leishmaniasis [ML]) have been identified, and all of them cause tegumentary leishmaniasis (TL) (mucosal and cutaneous involvement) (1). In the last 20 years, the Pan American Health Organization (PAHO) has been notified of 1,067,759 cases of CL and ML in South America, and, in 2020, the Plurinational State of Bolivia was the fourth country in the area in terms of the number of cases notified (2). In Bolivia, TL is present in seven of the nine departments into which the country is administratively divided (La Paz, Pando, Beni, Cochabamba, Santa Cruz, Tarija, and Chuquisaca) (3). The largest numbers of reported cases are concentrated in the first four of the mentioned departments (4). *Leishmania* species identification in Bolivia is performed only sporadically, but the available data indicate that *L. braziliensis* is the most prevalent, being characterized in 90% of lesions in TL patients, and this is followed by L. braziliensis outlier (39%) and *L. lainsoni* (7%) (5, 6). Other species present in the country are L. guyanensis and L. amazonensis (7, 8). Recently, L. peruviana has also been detected (6).

The characterization of *Leishmania* species is important in disease management, as it allows for the administration of the appropriate treatment, facilitates the prediction of disease evolution, and provides epidemiological data on leishmaniasis (9, 10). Several biochemical (isoenzyme electrophoresis and isoelectrofocusing) and molecular techniques such as random amplified polimorphic DNA (RAPD), polymerase chain reaction-restriction fragment length polymorphism (PCR_RFLP), PCR- sequencing, multilocus sequence typing (MLST), multilocus microsatellite typing (MLMT), etc., have been applied for the identification of *Leishmania* species in different areas of the world (11–15). A widely used strategy is based on PCR amplification and the sequencing of the gene encoding a 70 kDa heat shock protein (*hsp70*) (13, 16, 17), which is highly conserved and occurs in multiple copies arranged in tandem (18).

Proteomic techniques, such as matrix-assisted laser desorption/ionization-time of flight mass spectrometry (MALDI-TOF MS) are currently being applied to characterize microorganisms (19, 20).

A MALDI-TOF MS technique to identify *Leishmania* spp. was first developed in 2014 (21–23). Due to its simplicity, speed in obtaining results, and low cost, MALDI-TOF MS could represent a suitable method for *Leishmania* characterization in countries with limited access to molecular tools but where the technique is already available in microbiology laboratories for the routine identification of bacteria and yeasts. Thus, the present study aimed to evaluate the usefulness of MALDI-TOF MS in the characterization of the *Leishmania* species causing TL in Bolivia, using *hsp70* gene sequencing as a reference technique.

## RESULTS

### Characterization via *hsp70* gene sequencing.

By sequencing the *hsp70* gene, we identified 55 strains as follows: 23 *L. braziliensis*, 18 *L. braziliensis* outlier, 1 *L. peruviana*, 1 *L. guyanensis*, 2 *L. lainsoni*, 2 L. mexicana and 1 L. amazonensis, as well as 2 possible hybrids of *L. braziliensis*/*L. peruviana*, and 5 of L. mexicana/L. amazonensis.

**FIG 1:**
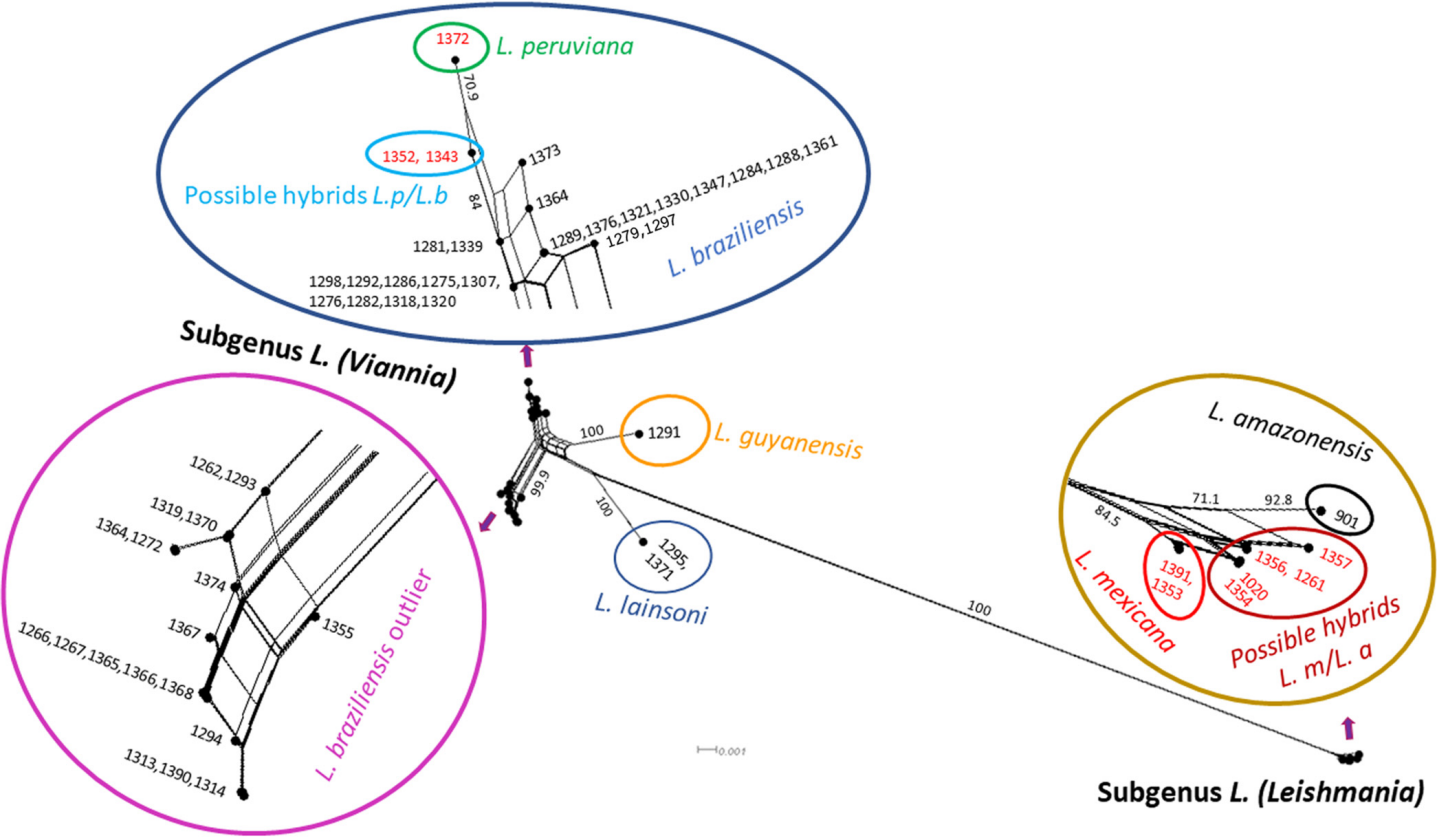
Genetic distance evaluation of *hsp70* by Neighbor-Net in *Leishmania* strains isolated from TL patients in Bolivia. The bootstrap values (1,000 replicates) are shown at the nodes (percentages), as are their relationships with the MALDI-TOF MS characterization results. The numbers in red correspond to the strains that were otherwise identified by MALDI-TOF MS, namely, *L. peruviana*, L. mexicana and hybrid strains of L. peruviana/L. braziliensis and *L mexicana*/L. amazonensis.

The genetic distances of the 55 strains that were analyzed via *hsp70* gene sequencing were evaluated via Neighbor-Net (NN) (Fig. 1). The networks showed a clear bifurcation between the subgenera *L.* (*Viannia*) and *L.* (*Leishmania*) (Bootstrap 100) as well as the division of the subgenus *L.* (*Viannia*) into three complexes: *L. braziliensis*, *L. lainsoni*, and *L. guyanensis* (Bootstrap 100). Within the *L. braziliensis* complex, *L. braziliensis* outlier was clearly distanced from the rest, and within *L. braziliensis* “sensu stricto,” a certain separation of *L. peruviana* was observed (Bootstrap 70.9). Within the subgenus *L.* (*Leishmania*), L. amazonensis was positioned separately from the other strains (Bootstrap 92.8), and the five possible hybrid strains of L. mexicana/L. amazonensis were located between L. mexicana and L. amazonensis (Bootstrap 66.2), but more closely to the former.

### MALDI-TOF MS.

MALDI-TOF MS identified the 55 strains as follows: 26 *L. braziliensis*, 18 *L. braziliensis* outlier, 1 *L. guyanensis*, 2 *L. lainsoni*, 5 L. amazonensis, and 3 *L. garnhami*. All of the isolates were correctly identified at the subgenus, genus, and complex level, but the comparison with the *hsp70* results indicated that 10 (18%) were misidentified as other species within the same complex. In 42 cases, the identifications were performed with confidence index A (76.4%), and in 13 cases, the identifications were performed with confidence index B (23.6%) (Table 1).

**TABLE 1:** Evaluation of the MALDI-TOF MS technique versus *hsp70* gene sequencing for the characterization of the *Leishmania* strains causing TL in Bolivia

Subgenus	Complex	*hsp70* gene sequencing	MALDI-TOF MS	
		Species (*n*)	Species (*n*)	Index
*L. (Viannia)*	*L. braziliensis*	*L. braziliensis* (23)	*L. braziliensis* (23)	A(16), B(7)
		*L. braziliensis* outlier (18)	*L. braziliensis* outlier (18)	A
		*L. peruviana* (1)	*L. braziliensis* (1)	A
		*L. braziliensis/L. peruviana* (2)^*a*^	*L. braziliensis* (2)	A
	*L. guyanensis*	*L. guyanensis* (1)	*L. guyanensis* (1)	B
	*L. lainsoni*	*L. lainsoni* (2)	*L. lainsoni* (2)	A
*L. (Leishmania)*	L. mexicana	L. mexicana (2)	L. amazonensis (2)	A
		L. amazonensis (1)	L. amazonensis (1)	B
		*L mexicana*/L. amazonensis (5)^*b*^	*L. garnhami* (3)	A(1), B(2)
			L. amazonensis (2)	B

a Possible hybrids: *L. braziliensis/L. peruviana*.

b Possible hybrids: L. mexicana/L. amazonensis.

The MALDI-TOF MS characterization technique correctly identified 81.8% of the strains when considering all of the analyzed strains, and it correctly identified 93.8% of the strains when excluding the potential hybrid strains. MALDI-TOF MS cannot identify potential *Leishmania* hybrids, whereas the analysis of the *hsp70* gene polymorphism can detect the genetic characteristics of both species.

## DISCUSSION

Among the great variety of techniques used to identify *Leishmania* species, isoenzyme characterization has long been considered the gold standard (24, 25). Nevertheless, due to the complexity of the isoenzyme technique, characterization is currently carried out mainly via molecular analysis, although this occurs without consensus on what constitutes the most suitable method or marker (25, 26).

MALDI-TOF MS is a proteomic technique that has proved highly useful for the identification of microorganisms, including bacteria, fungi, and parasites (27–30). First introduced in 1987, its developers were awarded with a Nobel Prize in 2002 (31). This technique is accurate and inexpensive, provides comprehensive information, and is fast and easy to handle (32, 33).

MALDI-TOF MS is now integrated into the daily routine work of many laboratories, greatly simplifying pathogen identification and improving patient care (34). Several well-established open access and commercial platforms based on MALDI-TOF MS are available for the identification of bacteria and yeasts. Most of the proprietary spectral databases can be customized by adding spectra to improve the discriminatory power of MALDI-TOF MS, including in strain typing (31).

The MALDI-TOF MS technique was first applied for *Leishmania* identification in 2014, with the creation of a library that can be accessed through a free, web-based application (21, 23). A commercial spectral library for *Leishmania* identification is still unavailable, and the methodology is mainly used in reference laboratories that either have their own databases or access the online library at https://msi.happy-dev.fr/ and apply their own interpretive criteria. Previous reports indicate that the identification of *Leishmania* strains via MALDI-TOF MS generates more discrepancies for New World species than for Old World species, with the exception of the L. donovani complex (13, 21, 22, 35).

The suitability of MALDI-TOF MS for the identification of *Leishmania* has been scarcely investigated to date. To our knowledge, two studies assessed its performance in routine practice, but they analyzed a small number of strains: a study carried out in France with 20 strains isolated from travelers with CL (*n* = 19) and VL (*n* = 1) (35) and another in Turkey, including 2 strains isolated from 2 patients with CL (22). Another comparative study of analytical methods, including MALDI-TOF MS, analyzed 53 strains isolated from autochthonous and imported cases of TL in Spain (36).

In our context, 55 *Leishmania* strains that were isolated from patients in Bolivia were analyzed via both MALDI-TOF MS and *hsp70* sequencing (the latter as a reference) to assess the usefulness of mass spectrometry in this context. The strains were isolated from TL patients in a Cochabamba laboratory that routinely performs culture-based diagnosis (37). Once the strains are obtained, the application of the MALDI-TOF technique is straightforward (35). In our study, all of the *Leishmania* strains were correctly identified at the subgenus and complex levels by MALDI-TOF MS. At the species level, 45 strains (81.8%) were correctly identified. Previous studies reported inconclusive results or misidentifications at the complex level for certain strains. Cassagne et al. (21) obtained uninterpretable results for two strains of L. major. Mouri et al. (35) could not differentiate between species of the *L. braziliensis* and *L. guyanensis* complexes, and similarly, there was one strain in Lachaud et al. (23) that was misidentified between these complexes. Recently, the MSI online application (used here and in Lachaud et al. [23]) has been improved by the incorporation of new reference strains and modifications to the algorithm. However, there are still identification problems with the *L. peruviana* and L. mexicana strains. Regarding the hybrid strains found in our study, all were identified as a single species. No studies were found concerning the analysis of hybrid strains via MALDI-TOF MS. When excluding the possible hybrids, we observed an increase of the correctly identified strains (93.8%). Comparable results have been obtained in previous studies, with their agreement percentages ranging from 86% to 95.65% (13, 21, 23).

Similar misidentifications of *Leishmania* species have also been reported. In a study in which 268 strains were analyzed via MALDI-TOF MS using MLEE, RFLP, and *hsp70* and *rpoIILS* gene sequencing as reference techniques (23), 31 of the strains (11.6%) were erroneously characterized. Discrepancies mainly arose in the identification of species within the *L. braziliensis* and *L. guyanensis* complexes from the New World and within the L. donovani complex from the Old World. In our study, the misidentification of *Leishmania* species by MALDI-TOF MS was 6/42 (14.3%) with confidence index A and 4/13 (30.8%) with confidence index B, which indicates that misidentification at the species level was independent of the confidence index and was more likely related to possible hybrid strains.

Among the strains identified with confidence index A, one *L. peruviana* isolate was characterized as *L. braziliensis*. The taxonomic position of *L. peruviana* in the *L. braziliensis* complex has been the subject of discussion (6, 24) and although both species are commonly misidentified, there is a stronger tendency for the ambiguous strains to be characterized as *L. peruviana* (13, 23). Additionally, two strains of L. mexicana were erroneously identified as L. amazonensis, another member of the L. mexicana complex. Misidentification at the species level in this complex is also frequent, and L. mexicana has been identified as L. pifanoi (and vice-versa) (23).

Of the 13 strains identified by confidence index B, a correct identification was found for *L. braziliensis* (*n* = 7), *L. guyanensis* (*n* = 1), and L. amazonensis (*n* = 1). In contrast, other studies have failed to correctly distinguish *L. guyanensis* from other species of the complex (23, 36). Misidentifications with confidence index B may be due to sample quality, parasite concentration, and the type of culture media used (35). However, the main issue is not inaccurate profiling, as reliable results have been obtained with MALDI-TOF MS for a wide spectrum of bacterial microorganisms (38). Instead, the main limitation of this system is its dependence on the quality and precision of the database, in which all organisms need to be represented equally, and the spectra, which should not be obtained solely from clinical strains (39). For example, the inaccurate identification of filamentous fungi by MALDI-TOF MS seems to be more related to the heterogeneity of protein profiles and their lack of representation in current databases (38). Similarly, for *Leishmania* species identification, the spectral library should be expanded to include strains from different regions that are endemic for leishmaniasis and, in particular, reference spectra from New World strains. Improving the software with additional data (27) would for allow the identification of less frequent species, as recommended for mycobacteria (32). Nevertheless, in the case of *L. lainsoni*, for which only two strains have been described, a correct identification was obtained, as in other studies (23).

MALDI-TOF MS represents a useful tool for the rapid and accurate characterization the of *L. braziliensis* outlier, which seems less likely to cause complications than does *L. braziliensis* “sensu stricto”. According to our results, none of these strains were isolated from ML, nor were they associated with therapeutic failures in treated patients (6).

Other probable explanations for the misidentifications and low confidence indices are related to genetics. When the results of MALDI-TOF MS are superimposed on the NN network, the strains of the subgenus *Leishmania* (*n* = 8) are clearly separated from those of the subgenus *Viannia* (Bootstrap 100). Also, *L. lainsoni* and *L. guyanensis* (Bootstrap 100) of the subgenus *Viannia*, as well as *L. braziliensis* outlier (Bootstrap 99.9), all form widely distanced clusters, and they were correctly identified by MALDI-TOF MS. On the other hand, less obvious genetic distances were observed between the *L. braziliensis* “sensu stricto” strains, the two possible *L. peruviana/L. braziliensis* hybrids, and even *L. peruviana* (Bootstrap 70.9), all of which were classified as *L. braziliensis* by MALDI-TOF MS.

Within the L. mexicana complex, excluding the hybrids, all of the strains (two L. mexicana and one L. amazonensis) were characterized by MALDI-TOF MS as L. amazonensis. These divergent results may be due to the high genetic and geographic variability between members of the complex, although this has not been comprehensively explored to date (24). Moreover, few strains belonging to the complex have been included in previous assessments of MALDI-TOF MS performance (21, 23, 35). Even so, in the NN network, the L. amazonensis strain identified by MALDI-TOF MS with index B appears to be separated from the other strains of the L. mexicana complex, displaying a bootstrap value of 92.8, which supports a correct identification at the species level.

The seven strains identified as possible hybrids in this study deserve particular attention. The two strains of *L. braziliensis*/*L. peruviana* and five strains of L. mexicana/L. amazonensis were identified by MALDI-TOF MS as species within the *L. braziliensis* and L. mexicana complexes, respectively. This limitation, namely, the lack of discrimination by MALDI-TOF MS, is possibly due to the variability of the *hsp70* gene sequence in the *L. braziliensis*/*L. peruviana* hybrid strains, which have been previously analyzed (6, 40, 41). We observed that the possible hybrid strains of the L. mexicana complex present 3 to 4 polymorphic sites in the *hsp70* gene (data not shown). Although this New World complex has been studied previously (24), no data about hybrids have been reported. Some Bolivian variants of the L. mexicana complex have been described based on *Cyt-b* gene and ITS-1 sequencing (8), and there is evidence of genetic exchange for L. amazonensis, both in the culture of axenic promastigotes and in infected macrophages, but not in humans (42). *Leishmania* hybrids have also been found between Old World species, such as L. aethiopica/L. donovani (43), L. infantum/L. donovani in vectors (44), and L. infantum/L. major in *in vitro* assays and *in vivo* infections (45). MALDI-TOF MS could also be limited for their identification.

MALDI-TOF has the disadvantage of requiring axenic cultures of parasites for species identification because, in most cases, the low number of microorganisms present in clinical samples does not allow for the obtaining of accurate spectra (39). It is expected that recent innovations, such as the incorporation of imaging mass spectrometry, will allow for the direct analysis of tissue sections in the near future (46). For now, according to our results, MALDI-TOF MS constitutes an alternative technique in clinical microbiology laboratories for the identification of TL-causing *Leishmania* species when parasites are cultured for parasitological diagnosis, as in ours. Once the parasite culture is obtained (7 to 21 days, in our case), the technique rapidly yields information, allowing patients to receive prompt and precise treatments. The PAHO guide recommendations for the treatment of CL indicate that if the most prevalent species in the region is known, treatment should be initiated according to the clinical condition and the availability of the medication, as well as in consideration of the risk-benefit balance (10). Also, it is strongly recommended to treat adult patients with pentavalent antimonials in cases of *L. braziliensis* or mucosal or mucocutaneous leishmaniasis, and it is recommended to treat adult patients with miltefosine in cases in which L. panamensis, L. mexicana, *L. guyanensis*, and *L. braziliensis* are implicated. In Bolivia, treatment is administered regardless of the parasite species, and only the patient follow-up after treatment is complex-dependent. The first-line treatment is done with antimonials, except for special cases of mucosal leishmaniasis, in which amphotericin B is administered (3). In this study, the predominant species was *L. braziliensis*, and other species of the *L. braziliensis*, *L. guyanensis*, and L. mexicana complexes were identified. Thus, identification at the complex level remains adequate, as mentioned (23).

As mentioned, a limitation of the technique is the nondetection of possible hybrids that exist in certain areas, including in Bolivia, leading to the misidentification of species within the same complex. Nevertheless, MALDI-TOF MS offers considerable advantages over characterization techniques that are based on molecular biology. Although the equipment involves a large initial investment, it can be used in different areas of microbiology and significantly reduces the time required to obtain results and the costs of tests. Therefore, MALDI-TOF MS would constitute a suitable tool for the characterization of *Leishmania* species in countries of endemicity, once access to the free online database becomes available. In Bolivia, its application would allow for the improvement of the management of the follow-up of patients, the updating of the epidemiological data of the *Leishmania* species, and a contribution to the control of tegumentary leishmaniasis. In Latin American countries, it could help patients receive a specific treatment when this is included in the existing guides, as recommended by the Pan American Health Organization.

## MATERIALS AND METHODS

### *Leishmania* isolates.

A total of 55 *Leishmania* strains that were isolated from Bolivian patients in the region of Cochabamba (Bolivia) between 2004 and 2015 were used in this study. The isolates were cryopreserved at −80°C in the Laboratory of Parasitology at the Universidad Mayor de San Simón (UMSS) in Bolivia, and they were then sent to the Parasitology Laboratory at the University of Barcelona (UB) in Spain, where the strains were cryopreserved in the UB Trypanosomatid Cryobank until analysis.

Promastigotes were recovered by thawing the tubes containing the isolates in a water bath at 37°C and culturing in Schneider’s medium (Sigma-Aldrich, UK) supplemented with 20% fetal bovine serum (Life Science Production, Brazil) and 1% sterile human urine until the exponential phase of growth was reached (3 × 10^6^ promastigotes/ml) (23).

### *hsp70* gene sequencing.

Characterization via *hsp70* gene sequencing was performed as a reference technique in the Parasitology Laboratory of the UB (Barcelona, Spain). Using the commercial QIAmp DNA Minikit (Qiagen, Germany), DNA was extracted from 200 μL of culture in PBS, which was treated with 20 μL of proteinase K, following the manufacturer’s instructions.

The amplification of the 1,245 bp *hsp70* gene was performed by means of two PCRs, PCR-N (552 bp) and PCR-T (723 bp), which together covered the entire fragment, according to the protocol described in Torrico et al. (6). The PCR products were purified using EXOSAP-IT (Affymetrix USB, USA), and they were sequenced using the Sanger method at the Scientific and Technological Center of the UB (Barcelona, Spain). The identification of the *Leishmania* species is based on the comparison of the sequences under study with the reference sequences of the different species available in GenBank.

Neighbor-Nets (NN) were generated from *hsp70* data sets using the Splitstree software package, version 4.14.8 (47), using the uncorrected p method and an equal angles representation. The results of the *Leishmania* species characterization via MALDI-TOF MS were also included in the network to evaluate its usefulness for the identification of the *Leishmania* species that cause TL in Bolivia, compared to the reference method.

### MALDI-TOF MS.

The MALDI-TOF MS analysis was carried out in the Microbiology Laboratory of the Hospital de la Santa Creu i Sant Pau (HSCSP, Barcelona, Spain). A volume of 5 mL of exponential-phase promastigote culture was consecutively washed three times with 0.9% saline solution via centrifugation for 10 min at 1,690 × *g*. The last sediment obtained was resuspended in 20 μL of 0.9% saline solution. Immediately, 1 μL of each strain suspension (6 replicates per strain) was placed in the wells of the MALDI-TOF MS steel plate and homogeneously distributed using a toothpick. Once dried, each well was covered with 1 μL of matrix, α-cyano-4-hydroxycinnamic acid (Matrix HCCA Brucker, Bremen, Germany) and was allowed to dry again. 1 μL of Bacterial Test Standard (BTS) (Brucker, Bremen, Germany) was placed in the last well of the plate to validate the test in each run. Once the entire plate was dried, it was placed on the equipment support for reading. The mass spectra were acquired using an Autoflex II TOF-TOF instrument (Bruker Daltonics) with the Flex Control program, version 3.4 (Bruker Daltonics), using the default parameters (36).

The spectra that were obtained were uploaded to the Mass Spectra Identifying (MSI) application, an online spectrum library that is available at https://msi.happy-dev.fr/, for identification. The protein profile spectra of our isolates were compared to those in the MSI online database, which compiles reference spectra for many *Leishmania* species. The application compares the values of the peaks obtained from the problem spectra with those of strains in the database, using its own algorithms, and it returns the identification and three similarity values with respect to: (i) the most similar spectrum in the library (first score), (ii) the most similar spectrum belonging to another species that is included in the same taxonomic complex as the first spectrum (second score), and (iii) the most similar spectrum for another species that is not included in that complex (3rd score). The similarity values range from 0 to 100, with the latter value indicating a perfect match. The application also provides an index that assesses the confidence of the identification: A, up to species level (first score of >22, second score of >20, and first–second of >8) or (first score of >20 but ≤22 and first–second of >2); B, up to genus level (first score of >22, second score of >20, and first–second of <8) or (first score of >20 but ≤22 and first–second score of <2), and C: irrelevant (no score >20). The characterization result was interpreted using the “best score” criterion, meaning that the identified species corresponds to that of the replica with the highest similarity value.

### Ethical aspects.

This research was carried out with isolates from patients with suspected TL who attended the LABIMED (Cochabamba, Bolivia) as part of a collaborative research project between ISGlobal (Barcelona, Spain) and the CEADES Foundation (Cochabamba, Bolivia), together with other Bolivian isolates that were stored in the cryobank of Trypanosomatids UB. The techniques used for the diagnosis of TL are part of the routine medical care in the health center in Cochabamba. The study protocol was approved in Spain by the Ethics Committees of the Hospital Clínic de Barcelona (HCB/2014/0582) and in Bolivia by both the CEADES Salud y Medio Ambiente and the Facultad de Medicina UMSS. All of the patients with suspected leishmaniasis provided written informed consent (parents or guardians, in cases in which the patient was under 18 years of age) before participating in the study. All of the suspected cases of TL were diagnosed free of charge, and confirmed cases were referred for treatment.

### Data availability.

The sequences were submitted to the GenBank databases under the accession numbers MW507486–MW507526 and OP561794–OP561807 (http://www.ncbi.nlm.nih.gov).
